# Supplementary material for: Antibody prophylaxis may mask subclinical SIV infections in macaques
Source: Nature. 2025 Feb 5;639(8053):205–13. doi: 10.1038/s41586-024-08500-y (PMC11882457; doi:10.1038/s41586-024-08500-y)
Supplement: Supplementary file 1 — Supplementary Tables 1–6 and references. [file 41586_2024_8500_MOESM1_ESM.pdf]

nature portfolio

https://doi.org/10.1038/s41586-024-08500-y

Supplementary information

Antibody prophylaxis may mask subclinical SIV infections in macaques

In the format provided by the authors and unedited

Table of Contents

Supplementary Tables	Page
**Table 1.** Study mAb neutralization IC50 and IC80 titers against SIV isolates	3
**Table 2.** Control group barcode sequencing	4
**Table 3.** ITS102.03 group barcode sequencing	5
**Table 4.** ITS103.01 group barcode sequencing	6
**Table 5.** Partially/fully neutralizing mAb infusion and challenge study animal demographics	7
**Table 6.** Barcoded virus challenge study animal demographics	8
**References**	9

Supplementary Table 1. Study mAb neutralization IC50 and IC80 titers against SIV isolates

Neutralization curves are shown in Extended Fig. 1A.

	Virus					
	SIVsmE660.A8.CP3C		SIVsmE660.2A5.CR54		SIVmac239	
**mAb**	**IC50**	**IC80**	**IC50**	**IC80**	**IC50**	**IC80**
ITS01	0.013	0.046	0.154	>50	>50	>50
ITS06.02	0.016	>50	>50	>50	>50	>50
ITS102.03	0.072	0.244	0.228	0.879	0.867	3.0
ITS103.01	0.015	0.052	0.017	0.052	0.007	0.022

Supplementary Table 2. Control group barcode sequencing

Barcodes are colored according to legend in Fig. 4A.

Animal	Plasma timepoint (Days post first challenge)	Dominant barcode
B1-1	18	A
	28	A
	42	A
B1-2	18	A
	28	A
	42	A
B1-3	18	A
	28	A
	42	A
B1-4	18	A
	28	A
	42	A

Supplementary Table 3. ITS102.03 group barcode sequencing

Barcodes are colored according to legend in Fig. 4A.

Animal	Plasma timepoint (Days post first challenge)	Dominant barcode	Other barcodes and abundance (percent of total reads)
B2-1	18	A	
	21	A	
B2-2	21	B	
	32	B	
	39	B	
	42	B	
B2-3	21	A	
B2-4	21	B	
	32	B	
B2-5	18	B	
	21	B	
	32	B	C (0.02)
B2-6	21	A	
	32	A	

Supplementary Table 4. ITS103.01 group barcode sequencing

Barcodes are colored according to legend in Fig. 4A. “N/R” indicates that no barcode sequences were able to be recovered at a given timepoint.

Animal	Plasma timepoint (Days post first challenge)	Dominant barcode	Other barcodes and abundance (percent of total reads)
B3-1	46	G	
	49	G	
	53	G	
	56	G	H (0.5)
	60	G	H (0.08), A (0.01)
	63	G	H (0.1)
	67	G	H (0.07)
B3-2	35	N/R	
	63	N/R	
	71	A	E (20)
	77	A	B (0.038)
	77	A	B (0.023)
	84	A	B (0.037)
B3-3	49	N/R	
	67	A	
	71	A	I (0.04)
	74	A	
	77	A	
	81	A	
	84	A	
B3-4	35	F	
	56	H	
	67	H	
	63	H	I (0.0015)
	67	H	
	71	H	
	74	H	
	77	H	
B3-5	35	N/R	
	67	A	
	67	A	
	71	A	G (0.001)
	74	A	
	77	A	
	81	A	
B3-6	35	N/R	
	60	I	A (12)
	67	I	A (16)
	71	I	A (39), F (0.0026), E (0.0008)

Supplementary Table 5. Partially/fully neutralizing mAb infusion and challenge study animal demographics

M = male, F = female. Age and weight were recorded at the start of the study. “TFP” denotes *TRIM5* alleles 1–5, “Q” denotes alleles 6–11, and “Cyp” indicates allele 12[1]. Animals that expressed MHC alleles (associated with control of virus replication[2,3]) *Mamu-A*01*, *Mamu-B*08*, or *Mamu-B*17* are denoted by “+” signs.

Animal	Sex	Age (years)	Weight (kg)	Trim5α genotype	*Mamu-A*01*	*Mamu-B*08*	*Mamu-B*17*
G1-1	M	2.6	4.50	TFP/TFP	–	–	–
G1-2	M	4.6	6.44	TFP/Q	–	–	+
G1-3	M	4.7	6.70	TFP/Q	–	–	–
G1-4	M	5.0	7.80	TFP/Q	–	–	–
G1-5	M	2.7	4.26	TFP/TFP	–	–	+
G1-6	F	5.0	6.77	Q/Cyp	–	–	–
G2-1	M	5.0	7.14	Q/Q	–	–	–
G2-2	M	4.6	6.46	TFP/Q	–	–	–
G2-3	M	5.0	8.56	TFP/TFP	–	–	–
G2-4	M	4.7	8.84	TFP/Q	–	–	–
G2-5	M	5.0	5.84	TFP/Q	–	–	–
G2-6	F	5.0	4.76	TFP/TFP	–	–	–
G3-1	M	4.7	8.56	TFP/TFP	–	–	–
G3-2	M	4.8	8.00	TFP/Q	–	–	–
G3-3	M	4.8	7.50	TFP/Q	–	–	+
G3-4	M	3.0	4.08	TFP/Q	+	–	–
G3-5	F	5.0	7.46	TFP/TFP	–	–	–
G3-6	F	5.0	7.40	Q/Q	–	–	–
G4-1	M	5.0	8.26	TFP/Q	–	–	–
G4-2	M	4.7	7.04	TFP/Q	–	–	–
G4-3	M	5.0	9.92	TFP/Q	–	–	–
G4-4	M	5.0	7.86	TFP/TFP	–	–	–
G4-5	M	3.0	4.20	TFP/Q	–	–	–
G4-6	F	4.7	5.92	TFP/Q	–	–	–
G5-1	M	4.8	8.78	TFP/Q	–	–	–
G5-2	M	2.8	5.10	TFP/Q	–	–	–
G5-3	M	4.6	7.96	TFP/Q	–	–	+
G5-4	M	5.0	7.62	TFP/Q	–	–	+
G5-5	M	5.0	7.60	TFP/TFP	–	–	–
G5-6	F	5.0	5.58	TFP/Q	–	+	–

Supplementary Table 6. Barcoded virus challenge study animal demographics
M = male, F = female. Age and weight were recorded at the start of the study.

Animal	Sex	Age (years)	Weight (kg)
B1-1	M	2.8	3.62
B1-2	M	2.7	3.82
B1-3	M	2.9	3.9
B1-4	M	2.7	4.54
B2-1	M	2.8	3.76
B2-2	M	2.8	3.62
B2-3	M	3.0	5.08
B2-4	M	3.0	3.92
B2-5	M	2.5	3.7
B2-6	M	2.7	4.18
B3-1	M	2.6	3.66
B3-2	M	2.9	4.92
B3-3	M	2.9	4.02
B3-4	M	3.0	4.0
B3-5	M	2.8	3.72
B3-6	M	2.8	3.88

References

1 Lim, S. Y. *et al.* TRIM5α Modulates Immunodeficiency Virus Control in Rhesus Monkeys. *PLoS pathogens* **6**, e1000738 (2010).

2 Zhang, Z. Q. *et al.* Mamu-A*01 allele-mediated attenuation of disease progression in simian-human immunodeficiency virus infection. *J Virol* **76**, 12845-12854 (2002). https://doi.org/10.1128/jvi.76.24.12845-12854.2002

3 Martins, M. A. *et al.* Mamu-B*17(+) Rhesus Macaques Vaccinated with env, vif, and nef Manifest Early Control of SIVmac239 Replication. *J Virol* **92** (2018).
