# Supplementary material for: Antibody prophylaxis may mask subclinical SIV infections in macaques
Source: Nature. 2025 Feb 5;639(8053):205–13. doi: 10.1038/s41586-024-08500-y (PMC11882457; doi:10.1038/s41586-024-08500-y)
Supplement: Supplementary file 2 — Reporting Summary [file 41586_2024_8500_MOESM2_ESM.pdf]

nature portfolio

Corresponding author(s): Mario Roederer

Last updated by author(s): Nov 6, 2024

Reporting Summary

Nature Portfolio wishes to improve the reproducibility of the work that we publish. This form provides structure for consistency and transparency in reporting. For further information on Nature Portfolio policies, see our Editorial Policies and the Editorial Policy Checklist.

Statistics

For all statistical analyses, confirm that the following items are present in the figure legend, table legend, main text, or Methods section.

n/a	Confirmed	
☐	☒	The exact sample size (*n*) for each experimental group/condition, given as a discrete number and unit of measurement
☐	☒	A statement on whether measurements were taken from distinct samples or whether the same sample was measured repeatedly
☐	☒	The statistical test(s) used AND whether they are one- or two-sided<br>*Only common tests should be described solely by name; describe more complex techniques in the Methods section.*
☒	☐	A description of all covariates tested
☐	☒	A description of any assumptions or corrections, such as tests of normality and adjustment for multiple comparisons
☐	☒	A full description of the statistical parameters including central tendency (e.g. means) or other basic estimates (e.g. regression coefficient) AND variation (e.g. standard deviation) or associated estimates of uncertainty (e.g. confidence intervals)
☐	☒	For null hypothesis testing, the test statistic (e.g. *F*, *t*, *r*) with confidence intervals, effect sizes, degrees of freedom and *P* value noted<br>*Give P values as exact values whenever suitable.*
☒	☐	For Bayesian analysis, information on the choice of priors and Markov chain Monte Carlo settings
☒	☐	For hierarchical and complex designs, identification of the appropriate level for tests and full reporting of outcomes
☒	☐	Estimates of effect sizes (e.g. Cohen's *d*, Pearson's *r*), indicating how they were calculated

Our web collection on statistics for biologists contains articles on many of the points above.

Software and code

Policy information about availability of computer code

Data collection	ELISA data was collected on a SpectraMax 384 Plus Absorbance Plate Reader (Molecular Devices)<br>Flow cytometry data was collected on a BD FACSymphony flow cytometer.<br>Neutralization data was collected on a SpectraMax L luminometer (Molecular Devices).<br>For single genome amplification, amplicons were Sanger sequenced on a 3730XL DNA Analyzer (Thermo Fischer Scientific).<br>Next-generation sequencing was performed using a MiSeq instrument (Illumina).
Data analysis	Data analysis was performed in GraphPad Prism v10.0.3 and JMP 15.<br>Flow cytometry data was analyzed using using FlowJo version 10.8.2 (Treestar, Inc., Ashland, OR).<br>Single genome amplification sequences were aligned using Geneious Prime software version 2023.0.<br>Sequence analysis was performed using the Frederick National Laboratory for Cancer Research Barcode Analysis Tool version 2022 (https://frederick.cancer.gov/research/aids-and-cancer-virus-program/sections/retroviral-evolution-section OR https://github.com/KeeleLab?tab=repositories), a custom algorithm written in R (version 4.3.1) to analyze barcoded viruses and MiSeq Env.

For manuscripts utilizing custom algorithms or software that are central to the research but not yet described in published literature, software must be made available to editors and reviewers. We strongly encourage code deposition in a community repository (e.g. GitHub). See the Nature Portfolio guidelines for submitting code & software for further information.

Data

Policy information about availability of data

All manuscripts must include a data availability statement. This statement should provide the following information, where applicable:

- Accession codes, unique identifiers, or web links for publicly available datasets
- A description of any restrictions on data availability
- For clinical datasets or third party data, please ensure that the statement adheres to our policy

The single genome sequence data have been deposited in GenBank with the accession codes PP458374 - PP458484 (http://www.ncbi.nlm.nih.gov/nuccore/). The MiSeq data have been deposited with links to BioProject accession number PRJNA1086015 in the NCBI BioProject database (https://www.ncbi.nlm.nih.gov/bioproject/). The other data that support the findings of this study are available in the source data document or from the corresponding author upon reasonable request.

Research involving human participants, their data, or biological material

Policy information about studies with human participants or human data. See also policy information about sex, gender (identity/presentation), and sexual orientation and race, ethnicity and racism.

Reporting on sex and gender	Not applicable
Reporting on race, ethnicity, or other socially relevant groupings	Not applicable
Population characteristics	Not applicable
Recruitment	Not applicable
Ethics oversight	Not applicable

Note that full information on the approval of the study protocol must also be provided in the manuscript.

Field-specific reporting

Please select the one below that is the best fit for your research. If you are not sure, read the appropriate sections before making your selection.

☒ Life sciences ☐ Behavioural & social sciences ☐ Ecological, evolutionary & environmental sciences

For a reference copy of the document with all sections, see nature.com/documents/nr-reporting-summary-flat.pdf

Life sciences study design

All studies must disclose on these points even when the disclosure is negative.

Sample size	Sample size was determined using power calculations for resolving differences between two means (independent samples t test) using a type I error rate of 0.05 and estimated average times to infection (in weeks) using historical data. For the first animal study, control animals were assumed to become infected on average at the first challenge (SD = 1) and mAb-treated animals would have a 2 week delay to infection (SD = 1). For a minimum of 85% power, 6 animals were required per group (power = 87.6%). Results from the first study were then used to inform estimates in power calculations for the second study. Using the relative difference in ITS103.01 and ITS102.03 in vitro IC80 values, 6 animals in each mAb infusion group was determined to provide >95% power to detect a difference in the time to infection between groups 2 and 3. For a minimum of 85% power, 4 animals in the control group were calculated to be sufficient (provides >95% or 91% power to detect a difference with ITS103.01 or ITS102.03 groups, respectively).
Data exclusions	Calculation of an ITS01 half-life for animal G4-3 is not shown due to high endogenous reactivity to the ITS01 anti-idiotypic antibody preventing calculation of mAb decay curve.
Replication	Animal studies were not replicated. Viral load measurements were generally not replicated given the limited amount of plasma available from the frequent blood sampling schedule utilized, however where practical samples were run in duplicate or triplicate. Plasma infused antibody measurement was repeated for a given sample at 2-3 different dilutions and an average value calculated. Neutralization and Env binding assays were performed in duplicate or triplicate. Intracellular cytokine staining was not replicated given the limited number of cells available at most time points. Sequencing was generally not replicated, except for repeated attempts at determining viral barcodes from viral blips.
Randomization	Animals were balanced into experimental groups on the basis of age, weight, sex, and (where required) TRIM5a genotype. The samples collected from these animals and used in the assays reported in the manuscript were allocated according to the animal/group from which they were derived.
Blinding	Investigators were not blinded to the treatments the animals received as they coordinated the study. Viral load testing and sequencing was performed with investigators blind to group allocation during data collection and initial analysis. Antibody measurements were not performed

blind due to the substantial time and cost involved with assaying all animals for all potential infused mAbs at all timepoints. ICS was performed blind to the animal group allocation.

Reporting for specific materials, systems and methods

We require information from authors about some types of materials, experimental systems and methods used in many studies. Here, indicate whether each material, system or method listed is relevant to your study. If you are not sure if a list item applies to your research, read the appropriate section before selecting a response.

Materials & experimental systems

n/a	Involved in the study
☐	☒ Antibodies
☐	☒ Eukaryotic cell lines
☒	☐ Palaeontology and archaeology
☐	☒ Animals and other organisms
☒	☐ Clinical data
☒	☐ Dual use research of concern
☒	☐ Plants

Methods

n/a	Involved in the study
☒	☐ ChIP-seq
☐	☒ Flow cytometry
☒	☐ MRI-based neuroimaging

Antibodies

Antibodies used

For intracellular cytokine staining (see OMIP referenced in methods section for concentrations used):
Antibody, Supplier, Clone number, Lot number
CD3 APC-Cy7, BD Biosciences, SP34.2, 1152687
CD4 PE-Cy5.5, Thermo Fisher, SK3, 2516573
CD8 BV570, BioLegend, RPA-T8, B346256
CD45RA PE-Cy5, BD Biosciences, 5H9, 8200578
CCR7 BV650, BioLegend, G043H7, B340645
CXCR5 PE, Thermo Fisher, MU5UBEE, 2404260
CXCR3 BV711, BD Biosciences, 1C6/CXCR3, 0309602
PD-1 BUV737, BD Biosciences, EH12.1, 0303349
ICOS Pe-Cy7, BioLegend, C398.4A, B293719
CD69 ECD, Beckman Coulter, TP1.55.3, 7620097
IFNg Ax700, BioLegend, B27, B320892
IL-2 BV750, BD Biosciences, MQ1-17H12, 2033660
IL-4 BB700, BD Biosciences, MP4-25D2, 1042139
TNF-FITC, BD Biosciences, Mab11, 1145433
IL-13 BV421, BD Biosciences, JES10-5A2, 1200672
IL-17 BV605, BioLegend, BL168, B338018
IL-21 Ax647, BD Biosciences, 3A3-N2.1, 1179052
CD154 BV785, BioLegend, 24-31, B329207

Anti-SIV mAbs:
Antibody, Supplier, Lot number
ITS01-LS, expressed in-house
ITS06.02-LS, expressed in-house
ITS103.01-LS, VPP, NTT627-160-04
ITS103.01, expressed in-house
ITS102.03, expressed in-house

Anti-idiotype mAbs:
Antibody, Supplier
17B4-IgG1 (anti-ITS01), expressed in-house
8A4-IgG1 (anti-ITS06.02), expressed in-house
anti-ITS103-Id1, expressed in-house
anti-ITS102-Id1, expressed in-house

Other antibodies:
Antibody, Supplier, Catalog No. (reactivity information)
Mouse Anti-Monkey IgG-HRP, SouthernBiotech, SB108a (Rhesus and cynomolgus IgG. Minimal reactivity to human and rabbit immunoglobulins, goat IgG, and mouse, rat, hamster, guinea pig, sheep, donkey, bovine, horse, porcine, feline, and chicken serum)
Goat Anti-Human Ig κ chain-HRP, Millipore Sigma, AP502P (Human. Reacts with human kappa light chains. Absorbed for human myeloma proteins with λ light chains and mouse immunoglobulins)

Validation

The ITS01, ITS06.02, ITS103.01, ITS102.03 mAbs were described in the manuscripts Mason et al, PLoS Pathogens 2016, PMID 27064278 and Welles et al, PLoS Pathogens 2022, PMID 35709309.
The ITS103.01-LS mAb was biochemically validated by the NIH VRC Vaccine Production Program. All ITS mAbs used for administration (ITS01-LS, ITS06.02-LS, ITS103.01-LS, ITS103.01, ITS102.03) were validated for purity by SDS-PAGE, antigen binding by ELISA assay, and activity by neutralization assay.

The anti-idiotype antibodies were described in the manuscripts Welles et al, PLoS Pathogens 2018, PMID 30517201 and Welles et al, PLoS Pathogens 2022, PMID 35709309 and validated by binding to their cognate antibody.
All other antibodies were commercially available products and subjected to routine testing by the supplying vendor.

Eukaryotic cell lines

Policy information about cell lines and Sex and Gender in Research

Cell line source(s)	293T (human cell line), female, ATCC<br>Expi293F (human cell line), female, ThermoFisher Scientific<br>TZM-bl (human cell line), female, NIH HIV Reagent Program
Authentication	Cell lines were all obtained from commercial sources/repositiories and were authenticated by the organizations from which they were obtained, specifically:<br>293T (ATCC) - STR profiling<br>Expi293F (ThermoFisher Scientific) - STR profiling and assessment of cell morphology/growth kinetics<br>TZM-bl (NIH HIV Reagent Program) - assessment of cell morphology and functional testing in neutralization assays
Mycoplasma contamination	Cell lines were not tested for mycoplasma contamination.
Commonly misidentified lines (See ICLAC register)	No commonly misidentified cell lines were used in this study.

Animals and other research organisms

Policy information about studies involving animals; ARRIVE guidelines recommended for reporting animal research, and Sex and Gender in Research

Laboratory animals	Forty-six research naïve, Indian origin rhesus macaques were used, aged between 2-5 years and weighing between 3-10kg.
Wild animals	No wild animals were used in this study.
Reporting on sex	In the first animal study, there were 7 females and 23 males. The females were distributed across the study groups as part of the randomization process. The second study used all male animals. Sex-based analysis was not performed due to the small study sample size.
Field-collected samples	No field-collected samples were used in this study.
Ethics oversight	All experiments were carried out in compliance with National Institutes of Health regulations and approval from the Animal Care and Use Committee of the Vaccine Research Center and Bioqual, Inc (Rockville, MD, USA), where NHPs were housed for the duration of the studies. Animals were housed and cared for in accordance with local, state, federal and institutional policies in facilities accredited by AAALAC International under standards established in the Animal Welfare Act and the Guide for the Care and Use of Laboratory Animals. In accordance with the institutional policies of both institutions, all compatible non-human primates are always pair-housed, and single housing is only permissible when scientifically justified or for veterinary medical reasons, and for the shortest duration possible. Non-human primates were housed in appropriately sized caging according to the Guide for the Care and Use of Laboratory Animals, eighth ed.40, and supplemented with a variety of enrichment toys, treats, fresh produce, and foraging devices. Water was offered ad libitum and animals were fed primate biscuits (Monkey Diet, 5038, Lab diet) twice daily. As standard practice, animal holding rooms were maintained on a 12-hour light/dark cycle, room temperature of 16–21°C, and relative humidity 30–70%.

Note that full information on the approval of the study protocol must also be provided in the manuscript.

Plants

Seed stocks	*Report on the source of all seed stocks or other plant material used. If applicable, state the seed stock centre and catalogue number. If plant specimens were collected from the field, describe the collection location, date and sampling procedures.*
Novel plant genotypes	*Describe the methods by which all novel plant genotypes were produced. This includes those generated by transgenic approaches, gene editing, chemical/radiation-based mutagenesis and hybridization. For transgenic lines, describe the transformation method, the number of independent lines analyzed and the generation upon which experiments were performed. For gene-edited lines, describe the editor used, the endogenous sequence targeted for editing, the targeting guide RNA sequence (if applicable) and how the editor was applied.*
Authentication	*Describe any authentication procedures for each seed stock used or novel genotype generated. Describe any experiments used to assess the effect of a mutation and, where applicable, how potential secondary effects (e.g. second site T-DNA insertions, mosiacism, off-target gene editing) were examined.*

Flow Cytometry

Plots

Confirm that:

☒ The axis labels state the marker and fluorochrome used (e.g. CD4-FITC).

☒ The axis scales are clearly visible. Include numbers along axes only for bottom left plot of group (a 'group' is an analysis of identical markers).

☒ All plots are contour plots with outliers or pseudocolor plots.

☒ A numerical value for number of cells or percentage (with statistics) is provided.

Methodology

Sample preparation	Cryopreserved PBMC from rhesus macaques were used for flow cytometry
Instrument	BD FACSymphony flow cytometer
Software	FlowJo version 10.8.2 (Treestar, Inc., Ashland, OR)
Cell population abundance	No cell sorting was performed
Gating strategy	For intracellular cytokine staining, single cells were identified based on FSC-A vs FSC-H, then live lymphocytes were identified using SSC-A vs Aqua blue viability stain. Cell were then gated as being CD3+, CD4 or CD8+, then as memory cells based on CD45RA- CCR7-, or CD45RA-CCR7+ or CD45RA+CCR7- prior to identifying cytokine positive cells based upon co-expression of the cytokine and CD69. Tfh-like cells were identified as CCR7+ CXCR5+ and PD-1+ ICOS+.

☒ Tick this box to confirm that a figure exemplifying the gating strategy is provided in the Supplementary Information.
